# How Do Clinicians Use Quotations in Goals of Care Notes?

**DOI:** 10.1016/j.chest.2025.01.014

**Published:** 2025-01-21

**Authors:** Gina M. Piscitello, Ruthe Ali, Katrina Hauschildt, Jane Schell

**Affiliations:** a Section of Palliative Care and Medical Ethics, Division of General Internal Medicine, University of Pittsburgh, Pittsburgh, PA; b Palliative Research Center, University of Pittsburgh, Pittsburgh, PA; c Department of Emergency Medicine, Cook County Hospital, Chicago, IL; d Division of Pulmonary and Critical Care Medicine, Johns Hopkins University School of Medicine, Baltimore, MD

**Keywords:** clinical medical ethics, goals of care, health disparities, quotations

## Abstract

**Background:**

Quoting patients in electronic medical record (EMR) notes is controversial. Quotations may be used to promote accuracy in documentation. However, they also may be used to cast skepticism on patient speech. Little is known about how quotations are used in EMR notes documenting goals-of-care (GOC) conversations.

**Research Question:**

How often are quotations used in GOC notes, what content do clinicians quote, and how does quotation use vary by clinician specialty and patient sociodemographic characteristics?

**Study Design and Methods:**

This multihospital, cross-sectional study assessed quotation use in GOC notes for seriously ill adult patients hospitalized between July and October 2021. Quotation frequency was evaluated and thematic analysis was used to assess the content of language quoted in GOC notes. The odds of quotation use by clinician specialty and patient sociodemographic group were determined using multivariable logistic regression.

**Results:**

Our review of 1,003 GOC notes across 14 hospitals found that quotations were used in 32% of notes and were used more often by palliative clinicians when compared with nonpalliative clinicians (38% vs 21%; unadjusted OR, 2.34 [95% CI, 1.74-3.14]; adjusted OR, 2.62 [95% CI, 1.66-4.13]). Quotations were present more often in notes of Black vs White patients (41% vs 30%; unadjusted OR, 1.61 [95% CI, 1.08-2.38]; adjusted OR, 1.73 [95% CI, 1.11-2.71]). The content of language included in quotations most often detailed patient feelings, family preferences, and patient discussion about death.

**Conclusions:**

This multicenter study found that quotations were used in almost one-third of GOC notes, were used more often by palliative vs nonpalliative clinicians, and were present more often in notes for Black vs White patients. Future research must explore clinician intentions in using quotations and identify whether quotation use may contribute to racial disparities in patient care.

## Graphical Abstract

**Figure:**
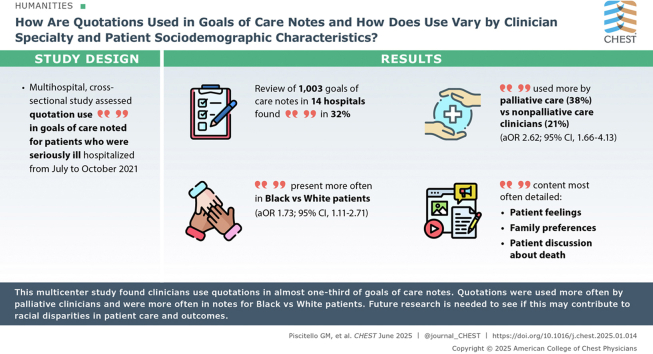

**Take-Home Points**

**Study Question:** How are quotations used in goals-of-care (GOC) notes and how does quotation use vary by clinician specialty and patient sociodemographic characteristics?

**Results:** Our multihospital review of 1,003 GOC notes found that quotations were used in about one-third of GOC notes, were used more often by palliative vs nonpalliative clinicians, and were present more often in notes of Black vs White patients.

**Interpretation:** This multicenter study found that clinicians use quotations more often in notes for Black vs White patients, which may contribute to racial disparities in patient care and outcomes.

Goals-of-care (GOC) conversations for hospitalized patients with serious illness are associated with improved patient quality of life, patient and family satisfaction, and quality of communication.^1^, ^2^, ^3^ These conversations among clinicians, patients, and surrogates clarify and align patient values and preferences with medical treatment options.^4^, ^5^, ^6^ Although GOC conversations for hospitalized patients increasingly are documented in the electronic medical record (EMR),^7^, ^8^, ^9^, ^10^, ^11^, ^12^ understanding about how clinicians document the content of these conversations is limited.

GOC conversations often include personal and sensitive information, such as what a patient values as they near the end of life and who they trust to make medical decisions on their behalf. Given that the content of these conversations may affect the patient’s clinical course, it is critical that these conversations are documented accurately in EMR notes. One tool that can be intended to promote accuracy in GOC documentation is the use of quotations. Clinicians can use quotations to document a patient's or surrogate’s speech directly, rather than documenting their own interpretation of this speech.^13^ However, use of quotations in GOC notes may impose potential harm to patients because clinicians also can use quotations to project doubt or skepticism on a patient's or surrogate’s speech. For example, clinician documentation of “the patient is hoping for a ‘miracle’” can be a way to portray clinician skepticism of the patient’s understanding of their clinical status.^14^, ^15^, ^16^

In this multihospital study, we aimed to assess how clinicians use quotations in their documentation of GOC conversations in the EMR for hospitalized adult patients. Specifically, we aimed to evaluate (1) the frequency of quotation use in GOC notes, (2) the content of language quoted by clinicians in GOC notes, and (3) whether quotation use in GOC notes varied by clinician specialty and patient sociodemographic characteristics.

## Study Design and Methods

### Overview and Study Design

This multihospital, cross-sectional study assessed GOC notes for seriously ill hospitalized adult patients across 14 hospitals within 1 large medical system in the northeast United States. These hospitals include urban, academic, and quaternary care hospitals as well as rural and nonrural community hospitals. This research study was reviewed by the University of Pittsburgh institutional review board (Identifier: STUDY14040128) and was determined to meet regulatory requirements for exempt research. We followed the Strengthening the Reporting of Observational Studies in Epidemiology guidelines to describe our research methods and results.^17^

### Setting

We identified adult patients with > 30% risk of 90-day mortality admitted to 1 of 14 hospitals during the study period from July 2021 through October 2021.^9^ We identified the first GOC note documented in the EMR for each hospitalized patient during the study period to include in our analysis. We identified these notes by searching for completion of a GOC template note signed by a clinician within the EMR. Clinicians at the study hospitals were actively encouraged to document all GOC conversations in this easily identifiable and searchable GOC note template, which has been described previously.^12^

### Demographics

We collected demographic information of hospitalized patients including patient age, sex, race or ethnicity, primary insurance status, rural status, and Area Deprivation Index.^18^ For some variables, we combined data into other categories to create a large enough group to complete χ^2^ and multivariable logistic regression analyses. Patients self-reported their sex and race or ethnicity. For race or ethnicity, measures included Black, White, and other. The other category included patients who were Alaska Native, Asian, Pacific Islander, Native American, Hispanic, other (multiracial), and undefined. For primary insurance status, categories included Medicaid, Medicare, private, and other. The other category included patients with national insurance and patients who lacked health insurance. We collected patient severity of illness measures including Elixhauser score and ICU admission. We also collected patient outcomes including change in code status during GOC conversation and discharge location. For each note, we identified the profession of the note writer (physician, advanced practice provider, medical trainee, other). The other category included palliative nurses and social workers. We also identified whether the note writer was a member of the palliative medicine team.

### Assessment of Quotations

Quotations were identified by reviewing GOC notes for the use of words contained within either single (‘’) or double (“”) quotation marks. For the quantitative assessment, any notes including at least 1 use of quotations was identified as a note with quotation use. For the qualitative thematic analysis, 2 trained research team members (G. M. P. and R. A.) independently reviewed the content within quotation marks for the entire study sample. The coders iteratively developed a codebook using an inductive approach, and thematic saturation was achieved.^19^ One hundred percent of quotations were double coded. Codes were compared between reviewers, and coding discrepancies were resolved with consensus adjudication.^20^

### Statistical Analyses

We descriptively analyzed patient characteristics of the study sample using quantity and percent or mean and SD. We compared the odds of quotation use in GOC notes by clinician specialty and patient sociodemographic group in an unadjusted model and an adjusted model using multivariable logistic regression. The adjusted multivariable logistic regression model was adjusted for patient age, sex, race, insurance, Area Deprivation Index, rural residency status, clinician specialty type, and hospital location and was clustered by clinician who wrote the note. We compared the unadjusted odds of quotation content themes by patient race. We did not adjust for confounding factors when assessing quotation content themes by patient race because of low frequency of data in each content theme category. Quantitative analyses were conducted using R software (R Foundation for Statistical Computing) and GraphPad Prism version 3.6.2 software (GraphPad Software).^21^ Qualitative analyses were completed using NVivo (Lumivero).^22^

## Results

### Patient and Clinician Demographics

A total of 1,003 patients admitted to 1 of 14 hospitals between July 2021 and October 2021 had at least 1 documented GOC note in the EMR during hospital admission. Most patients with documented GOC notes were older than 65 years (73%). By race, patients were 12% Black, 85% White, and 3% other. Almost one-quarter of patients (23%) had an Area Deprivation Index in the highest quintile of deprivation. Thirty-nine percent of patients were admitted to an ICU, 25% died during admission, and 23% were discharged with hospice. Thirty-nine percent of patients underwent a change in code status and 18% underwent a change to comfort measures only status during the documented GOC meeting (Table 1). Black patients were less likely to undergo a documented change in code status (31% vs 37%; *P* = .002) or change to comfort measures only status (16% vs 21%; *P* = .002) during the GOC meeting when compared with White patients. Forty percent of GOC notes were documented by an attending physician, 40% were documented by an advanced practice provider, 20% were documented by a medical trainee, and < 1% were documented by a palliative nurse or social worker (e-Table 1). Sixty-two percent of notes were documented by palliative medicine clinicians (physician, advanced practice provider, nurse, or social worker).

**Table 1:** Patient Characteristics

Patient Characteristic	GOC Notes			*P* Value^a^
	All (N = 1,003)	Without Quotations (n = 686)	With Quotations (n = 317)	
Race				
Black	119 (12%)	69 (10%)	49 (15%)	**.02**
Other	27 (3%)	19 (3%)	8 (3%)	> .99
White	857 (85%)	597 (87%)	260 (82%)	**.04**
Sex				
Female	491 (49%)	344 (50%)	146 (46%)	.25
Male	512 (51%)	341 (50%)	171 (54%)	.22
Age, y				
> 65	735 (73%)	511 (74%)	225 (71%)	.25
≤ 65	268 (27%)	176 (25%)	92 (29%)	.28
Insurance				
Medicaid	78 (8%)	50 (7%)	28 (9%)	.45
Medicare	808 (81%)	561 (82%)	246 (78%)	.12
Other	30 (3%)	13 (2%)	8 (3%)	.49
Private	87 (9%)	57 (8%)	30 (9%)	.55
Area Deprivation Index^b^				
80-100	227 (23%)	156 (23%)	70 (22%)	.87
Resides in rural United States	201 (20%)	143 (21%)	58 (18%)	.40
Admitted to ICU	387 (39%)	255 (37%)	131 (41%)	.21
Charlson Comorbidity Index	3 (2)	3 (2)	3 (2)	.91
Documented change in care plan noted in GOC note				
Change in code status	387 (39%)	270 (40%)	116 (37%)	.44
Change code status to comfort measures only status	185 (18%)	144 (21%)	41 (13%)	**.002**
Change to time-limited trial	13 (1%)	10 (1%)	3 (1%)	.76
Change to pursue all life-prolonging treatments	32 (3%)	20 (3%)	12 (4%)	.45
No change in care plan noted in GOC note	546 (54%)	368 (54%)	178 (56%)	**.004**
Discharge location				
Home	255 (25%)	172 (25%)	83 (26%)	.76
Hospice	235 (23%)	164 (24%)	71 (22%)	.63
Facility	263 (26%)	170 (25%)	84 (26%)	.58
Died	249 (25%)	170 (25%)	79 (25%)	> .99

Data are presented as No. (%) or mean (SD). Boldface values indicate statistical significance. Percentages may not add to 100 because of rounding. GOC = goals-of-care.

a Notes with quotations vs notes without quotations.

b Ranges from 0 to 100, with 100 indicating areas of highest disadvantage.

### Frequency of Quotation Use

Quotations were identified within 32% of all documented GOC notes. Palliative clinicians were more likely to use quotations in GOC notes when compared with nonpalliative clinicians (38% vs 21%; unadjusted OR, 2.34 [95% CI, 1.74-3.14]; adjusted OR, 2.62 [95% CI, 1.66-4.13]). Assessing quotation use across patient sociodemographic categories, Black patients were more likely to have at least 1 quotation documented within a GOC note when compared with White patients (41% vs 30%; unadjusted OR, 1.61 [95% CI, 1.08-2.38]; adjusted OR, 1.73 [95% CI, 1.11-2.71]) (Table 2). No difference was found in quotation use by patient sex, age, insurance status, rural status, or Area Deprivation Index.

**Table 2:** Multivariable Logistic Regression Analysis Identifying Factors Associated With Quotation Use

Characteristic	OR (95% CI)	*P* Value
Race		
Black	1.73 (1.11-2.71)	**.0162**
Other	0.87 (0.44-1.72)	.6803
White	Reference	
Sex		
Female	Reference	
Male	1.17 (0.87-1.56)	.3015
Age, y		
> 65	0.99 (0.66-1.48)	
≤ 65	Reference	.9488
Insurance		
Medicaid	1.05 (0.50-2.19)	.9014
Medicare	0.95 (0.52-1.72)	.8579
Other	1.63 (0.70-3.80)	.2551
Private	Reference	
Area Deprivation Index^a^		
80-100	0.93 (0.60-1.44)	.7482
Resides in rural United States	0.90 (0.59-1.38)	.6276
Clinician type		
Nonpalliative	Reference	
Palliative	2.62 (1.66-4.13)	**< .0001**
Hospital location		
A	Reference	
B	3.61 (0.59-16.19)	.0941
C	0.16 (0.01-2.23)	.1733
D	< 0.01 (< 0.01-< 0.01)	**< .0001**
E	< 0.01 (< 0.01-< 0.01)	**< .0001**
F	0.78 (0.18-3.32)	.7367
G	0.32 (0.09-1.15)	.0806
H	0.81 (0.23-2.91)	.7466
I	1.20 (0.38-3.59)	.7829
J	1.05 (0.25-4.46)	.9521
K	1.25 (0.40-3.91)	.7008
L	1.03 (0.33-3.25)	.9611
M	0.20 (0.05-0.88)	**.0336**
N	0.81 (0.18-2.65)	.7879

Boldface values indicate statistical significance.

a Ranges from 0 to 100, with 100 indicating areas of highest disadvantage.

### Content of Quotations

The most frequent content quoted within GOC notes was (1) patient feelings, (2) family preferences, and (3) patient discussion about death (Table 3). Clinicians quoted patient feelings about topics including their symptoms, prognosis, and perceptions of clinician behavior (Table 4). Here is an example of a clinician quoting a patient’s feeling that her clinicians were avoiding her: When asked what her sense of where things are at currently she shared she wasn’t sure and "feels like they are hiding from me."

**Table 3:** Occurrence of Quotation Themes by Patient Race

Quotation Themes	All (N = 1,003)	White (n = 857)	Black (n = 119)	Other (n = 27)	Unadjusted OR (95% CI), Black vs White Race
Patient feeling	55 (5)	47 (5)	6 (5)	2 (7)	0.9 (0.3-2.2)
Family preference	53 (5)	40 (5)	13 (11)	0 (0)	**2.5 (1.3-4.8)**
Patient discusses death	45 (4)	42 (5)	3 (3)	0 (0)	0.5 (0.2-1.6)
Family describes patient	42 (4)	34 (4)	6 (5)	2 (7)	1.3 (0.5-3.1)
Patient understanding	38 (4)	32 (4)	6 (5)	0 (0)	1.4 (0.6-3.3)
Clinician description	20 (2)	20 (2)	0 (0)	0 (0)	NA
Religion or spirituality	20 (2)	14 (2)	5 (4)	1 (4)	2.6 (0.9-7.5)
Family understanding	18 (2)	15 (2)	3 (3)	0 (0)	1.5 (0.4-5.1)
Family concern	15 (1)	10 (5)	4 (3)	1 (7)	2.9 (0.9-9.5)
Patient concern	9 (1)	9 (1)	0 (0)	0 (0)	NA

Data are presented as No. (%) unless otherwise indicated. Boldface values indicate statistical significance. NA = not applicable.

**Table 4:** Examples of Quotation Use for Most Frequent Themes

Theme	Quotation
Patient feeling	• “Day after day it’s the same old thing, laying her[e] constantly, getting stabbed every which way.” • He wishes that people would just “leave him alone” and that he wants to go home and “be comfortable” with family. • Her sons know her wishes and they need to follow them “or I'll haunt them.” • When asked what her sense of where things are at currently she shared she wasn’t sure and “feels like they are hiding from me.” • The idea of longevity or even “more good days” did not appeal to her since she has trouble imagining any “good days.” • She worries about her liver however is also glad that she doesn’t feel any discomfort, only thing when she came around was that she felt “antsy,” and wanted the NGT out and [to] be able to eat. • Patient was reluctant to accept this news stating that he did not believe that he was aspirating and he “did just fine at home.”
Family preference	• Family in agreement to avoid furthering p[atien]t’s “suffering.” • He is not willing to “give up” on her yet, “she's still breathing, and wouldn't give up on me.” • If p[atien]t’s heart stopped in current state . . . wife would like to “give him a jump start” if that occurs. • Her “end goal” is to bring her mother home. She is not ready to make any “big decisions” today. • His goal is to take her home and get her strong and work for “a cure” and live as long as possible. • He says selfishly he would be ok if [s]he was “bedridden” but he could still be with her. • They expressed that they would like “everything done” to bring him back.
Patient discusses death	• “I have been praying to God to take me, I do not want to do this anymore, this is really hard.” • “Dying is easy, it’s the getting to the point of dying that is hard.” • “I want to die, and it is time to go.” • “Dying is not a pleasant idea”, then shrugged and added, “I am 88.” • “If I die, let me die.” • They told him he is “dying,” and he “isn't ready to accept that.” • He realizes that his time is “likely shorter than I want . . . I don’t think this is going to go well for me.”

Clinicians quoted family preferences within GOC notes, with many of the quotations in this category either describing family preferences for life-prolonging care (43%) or comfort-focused care (36%). An example of a quotation of a family member’s preference for life-prolonging care is: his goal is to take her home and get her strong and work for "a cure" and live as long as possible.

An example of a quotation of a family member’s preference for comfort focused treatment is: family in agreement to avoid furthering p[atien]t’s "suffering.”

Quotations also were used by clinicians to describe patients discussing their feelings about death. The following are examples from two GOC notes: “I have been praying to God to take me, I do not want to do this anymore, this is really hard.” and “dying is easy, it’s the getting to the point of dying that is hard.”

### Quotation Themes by Patient Race

Because GOC notes for Black patients were more likely to contain quotations than those for White patients, we assessed whether the content of language included in quotations quantitatively differed by patient race. Family preferences were more likely to be quoted in GOC notes for Black patients when compared to notes for White patients (11% vs 5%; unadjusted OR, 2.5; 95% CI, 1.3-4.8). Clinicians were statistically more likely to use quotations to document family preferences for life-prolonging care in the notes of Black vs White patients (6% vs 2%; unadjusted OR, 3.3; 95% CI, 1.3-8.2), but not for quotations documenting family preferences for comfort-focused care (3% vs 2%; unadjusted OR, 1.4; 95% CI, 0.4-4.7). No other racial differences in the frequency of content included in quotations were found.

## Discussion

In our review of 1,003 GOC notes for seriously ill patients across 14 hospitals, we found that quotations were used in about one-third of all notes and were used more frequently by palliative vs nonpalliative clinicians. The frequency of quotation use differed by race, with quotations more often identified in GOC notes for Black patients when compared to notes for White patients. Frequency of quotation use did not differ across other patient sociodemographic characteristics such as age, sex, and Area Deprivation Index. The content of language included in quotations most often detailed patient feelings, family preferences, and patient discussion about death. Clinicians were more likely to quote family preferences in GOC notes written for Black vs White patients, with no other racial differences in quotation content by patient race identified.

This study is the first to our knowledge to assess the frequency of quotation use in GOC notes and to assess for differences in frequency by clinician specialty and patient sociodemographic characteristics. Although our findings are similar to past research assessing quotation use in internal medicine clinic notes in that we identified more frequent use of quotations in notes for Black patients, we did not similarly find differences in quotation use by patient sex.^23^ Our study also identified the content of language included in quotations in GOC notes and assessed for differences in this content by patient race, which has not been examined previously.

We identified that quotations were more often used by palliative vs nonpalliative clinicians. Although the reasons for this difference in quotation use are unknown, it is possible this finding may be associated with national palliative care guidelines and education requirements that emphasize identifying patient values and preferences for medical care, which may encourage palliative clinicians to use quotations more often in an attempt to document these important statements accurately.^24^^,^^25^ It is also possible that palliative medicine specialists may be more likely to be consulted for complex GOC conversations where disagreement exists among clinicians, patients, and surrogates, thus prompting more frequent use of quotations as a means of distancing the care team from patient or surrogate speech.

Our research identified that clinicians use quotations with higher frequency in GOC notes for Black patients, even after adjusting for factors including patient sociodemographic characteristics and hospital location. The reasons for differences in quotation use by patient race and implications for patient care are not clear. Although racial disparities in GOC conversations are known to exist for Black patients, including lower rates of GOC documentation, higher perceived clinician difficulty in having these conversations, and lower clinician time spent during GOC meetings, racial differences in clinician use of quotations in documented GOC notes is a new finding.^26^, ^27^, ^28^, ^29^ If clinicians use quotations more often for Black patients with the intent to accurately report and elevate patient voices in the setting of known racial discrimination in health care, more frequent quotation use in the notes of Black patients may be a positive finding.^30^, ^31^, ^32^, ^33^ However if clinicians are using quotations in GOC notes to cast doubt or judgment on patient speech, more frequent use of quotations for Black patients would be discriminatory. Because patients in the United States have increasing access to their medical records, patients can easily view if clinicians quote their speech in GOC notes.^34^ In this setting, clinician use of quotations may help to avoid patient perceptions that a clinician’s GOC documentation misinterpreted patient speech, because the patient’s words are quoted directly. In contrast, if patients perceive that clinicians use quotations to cast doubt or skepticism on the sensitive information they or their families privately shared during a GOC conversation, this may contribute to potential harms such as fracturing patient trust in clinicians, causing clinician-patient conflict, and leading patients to avoid seeking medical care.

When assessing the content of quotations in GOC notes, we found that family preferences for life-prolonging care were quoted more often in GOC notes for Black patients when compared with those for White patients. Several reasons may exist for this difference. First, family preferences for life-prolonging care may have been shared more often in GOC meetings for Black patients because a greater proportion of Black families expressed preferences for life-prolonging care when compared with White families.^35^ Second, clinicians may have quoted this content in the notes of Black patients more often in an attempt to more clearly document and advocate for these preferences to be honored within a US health care system in which Black patients have been shown to receive life-prolonging treatments such as invasive mechanical ventilation and extracorporeal membrane oxygenation at lower rates than White patients.^36^^,^^37^ Third, and most concerning, is that clinicians may be more likely to disagree with preferences for life-prolonging care of Black patients, and thus more often quote this content in the notes of Black patients as a way to show disagreement or to distance themselves from these expressed preferences for care.^38^

Our study only assessed the frequency and content of quotations in GOC notes. It did not assess the intent behind clinicians’ use of quotations such as to promote accuracy in documentation or to cast doubt on patient speech, which must be assessed in future research. Another important area for future research is to evaluate clinicians’, patients’, and families’ perceptions about the use quotations in GOC notes. Even if clinicians’ intent with the use of quotations is positive, it is possible quotations may be interpreted negatively by other clinicians, patients, or family members who read these notes, adversely impacting patient care and relationships among clinicians, patients, and families and potentially contributing to racial disparities in patient care and outcomes.

### Study Limitations

Although this study assessed GOC notes from 14 different hospitals across urban, suburban, and rural settings, all GOC notes were written by clinicians practicing in 1 state and may not be generalizable to other areas of the United States. Overall, the total number of quotation themes in each assessed category was low, limiting our ability to detect statistical differences that may be present in a larger data set. We reviewed quotation use in GOC notes only for hospitalized patients with serious illness. Quotation use in GOC notes in outpatient settings may differ from inpatient settings and should be assessed in future research. Our study is limited in that the patient population assessed included few non-English-speaking patients. Clinicians may use quotations differently for non-English-speaking populations, which is important to evaluate in future work. This study was unable to compare the content that was discussed verbally during GOC conversations with the content clinicians chose to quote in GOC notes. This study also did not assess the intent of quotation use and the impact of quotations on patient care and outcomes.

## Interpretation

This multicenter study of GOC notes for seriously ill hospitalized patients found that quotations were used in almost one-third of GOC notes, were used more often by palliative vs nonpalliative clinicians, and were present more often in notes for Black vs White patients. Future research is needed to identify reasons for differences in quotation use by clinician specialty and patient race and to assess how these differences may influence clinician and family perceptions, patient care, and health disparities.

## Funding/Support

The authors have reported to *CHEST* that no funding was received for this study.

## Financial/Nonfinancial Disclosures

None declared.

## Supplementary Data

e-Online Data
