# Supplementary material for: How Do Clinicians Use Quotations in Goals of Care Notes?
Source: Chest. 2025 Jan 21;167(6):1737–45. doi: 10.1016/j.chest.2025.01.014 (PMC12202787; doi:10.1016/j.chest.2025.01.014)
Supplement: e-Online Data [file mmc1.docx]

Online Supplement

e-Table 1. Quotation Use by Clinician Type

|  | **All (N=1003)** | **Non-Palliative APP**  **(n=79)** | **Non-Palliative Physician**  **(n=100)** | **Medical Trainee**  **(n=198)** | **Palliative APP**  **(n=325)** | **Palliative Physician**  **(n=297)** | **Other Palliative Team Member (Nurse, Social Worker)**  **(n=5)** | **P-Value** |
| --- | --- | --- | --- | --- | --- | --- | --- | --- |
| **Use of Quotations in Goals of Care Notes,**  **Quantity (Percent)** | 317 (32%) | 14  (18%) | 24  (24%) | 41 (21%) | 131 (40%) | 105  (35%) | 2  (40%) | <0.0001 |

APP – Advanced Practice Provider
